# Palatal Rugae as a Discriminating Factor in Determining Sex: A New Method Applicable in Forensic Odontology?

**DOI:** 10.3390/dj11090204

**Published:** 2023-08-29

**Authors:** Andrea Trizzino, Pietro Messina, Fabio Massimo Sciarra, Stefania Zerbo, Antonella Argo, Giuseppe Alessandro Scardina

**Affiliations:** 1 Department of Surgical Oncological and Stomatological Disciplines, University of Palermo, 90127 Palermo, Italy; andrea.trizzino@community.unipa.it (A.T.); pietro.messina01@unipa.it (P.M.); fabiomassimosciarra@gmail.com (F.M.S.); 2 Department of Promotion of Health, Maternal-child, Internal Medicine and Specialist of Excellence “G. D’Alessandro” University of Palermo, 90127 Palermo, Italy; stefania.zerbo@unipa.it (S.Z.); antonella.argo@unipa.it (A.A.)

**Keywords:** palatal rugae, sex, forensic dentistry

## Abstract

The purpose of this study is a new method that can help to identify the sex through the study of palatal rugae, comparing sagittal sections of the hard palate using Cartesian coordinates and evaluating the assistance given by digital technology and its applicability in this method. In this study, 57 digital impressions were examined and divided in two groups based on sex. Results: 2223 impression sections were studied and 145 coordinates that were present with a frequency greater than 50% in one or both groups were obtained: 52 discriminating traits (DT) in the male group, 29 discriminating traits in the female group, and 64 common traits (CT). The DTs in the female group showed no statistically significant difference from the same coordinates in the male one (*p* = 0.832). Statistically significant differences were observed in the DTs in the male group compared to the same coordinates in the female group (*p* = 0.018). No statistically significant differences were observed in the frequency of DTs in both sexes (*p* = 0.056). Further research in forensic odontology is needed to determine its scientific certainty. It is certain that digital technology may one day be a valuable support for the forensic odontologist but to date the lack of dedicated and certified programs limits its reliability.

## 1. Introduction

Forensic odontology is a branch of dentistry that deals with the identification of the individual through the study of the oral cavity. It brings together not only purely dental knowledge, but also anthropology and forensic medicine.

The stomatognathic apparatus is a unique structure. Not only does it have relevant interindividual differences, but it is also resistant to various injuries of considerable magnitude, which makes it capable of giving us information such as age and sex. Generally speaking, it can enable us to identify the individual [1].

Identification of the individual is one of the main purposes of forensic odontology, and it finds excellent application in mass disasters, shipwrecks and violent deaths, charred bodies or bodies in an advanced state of decomposition. In the history of forensic odontology, it has proven to be relevant in some situations even if some doubts still remain [2].

The investigation activities of the forensic odontologist are based on available ante-mortem data, post-mortem data, and instrumental investigations: CT scan, cheiloscopy and palatal rugoscopy [3].

In the context of palatal rugoscopy, palatal rugae in forensic odontology are an important identification marker in edentulous individuals [4]. It has been observed that the hard palate and its rugae represent an extremely stable structure over time in charred or decaying skulls [5], thus lending itself to becoming an important tool in the identification of individuals. However, in order to identify individuals with certainty, databases are needed. In the absence of such databases, the most rational use of this method, according to the authors may be limited to distinguishing sex or possibly certain ethnicities which have remained isolated.

To date, there are conflicting opinions in literature with regard to the determination of sex through palatal rugoscopy. Studies done on sex identification through rugoscopy are based on quantitative and qualitative characteristics, it’s an opinion of the authors that give results which are probabilistic and certainly variable among different ethnic group and thus not universally applicable. It is important to underline how the use of palatal rugae as a sex discriminant is limited and that it must be used in combination with other morphological elements or specific exams.

The use of digital technology in the twenty-first century is increasingly imposing itself in both daily life and in professional activities. The question remains whether these innovations can be applicable in forensic practice and, in particular, whether they can be used by the figure of the forensic odontologist.

The purpose of this study, unlike other studies and classical palatal rugocopy, is to identify sex by highlighting common coordinates and discriminating coordinates in the two sexes through a new approach to palatal rugoscopy, comparing sagittal sections of the hard palate and evaluating the assistance given by digital technology and its applicability in this method.

## 2. Materials and Methods

In this study, 85 digital impressions of the upper arch and hard palate acquired in different occasions were examined. The impressions were acquired using Primescan (Dentsply Sirona, York, PA, USA), 20 with iTero (Align Technology, Risch-Rotkreuz, Switzerland). The subjects ranged in age from 13 to 27 years old.

Of the 85 impressions, 28 were discarded because they did not meet the study criteria. 57 subjects were examined: 27 male and 30 female. 

Inclusion criteria:

- Full set of permanent teeth, from 16 to 26;
- Completeness of the palate in the intraoral scan;
- Indication of the patient’s sex.

Exclusion criteria:

- Agenesis;
- Inserted elements;
- Dysembryogenic alterations;
- Current or previous orthodontic therapy;
- Surgery on the hard palate;
- Implant prostheses or crowns on natural abutments;
- Previous rapid palate expansion;
- Palate injuries of various kinds.

Once the impressions were acquired in .stl format, the palatal rugae, retroincisal papilla and palatal cusps of the second premolars were isolated using Meshmixer software (Version 3.5, Autodesk Inc., San Rafael, CA, USA), eliminating manually everything else in the scan. In addition, while maintaining the proportions of the structures, they were resized so that the interpremolar distance, calculated as the distance between the two palatal cusps, was 39 mm. This decision was made in order to spatially standardize the 3D models.

Then the impressions were aligned using the same software in order to subsequently import them to Geomagic^®^ Control X 14 software (3D System, Santa Clara, CA, USA).

**Figure 1:**
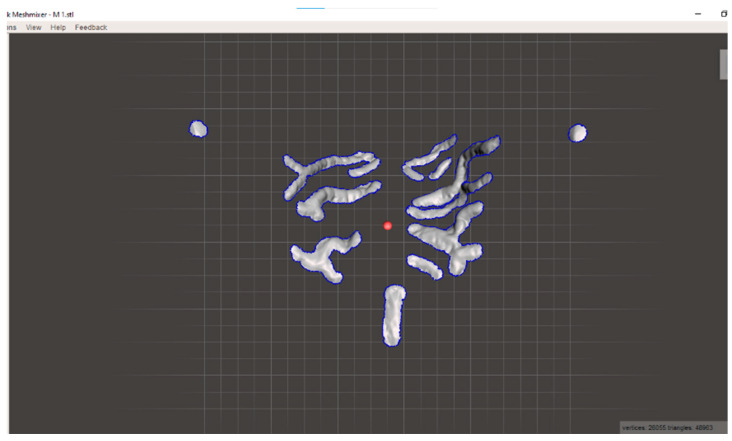
Isolated Model.

However, the Geomagic Control X software cannot give us the Cartesian coordinates of the impressions loaded onto it, and so a custom-made 3D grid was used to derive the coordinates of the ordinates (Figure 1).

The square-shaped 3D grid in question consists of 31 planes: 26 planes with 39 mm sides and 5 planes with 41 mm sides, all 1 mm apart. Planes 1, 6, 11, 16, 21, 26 and 31 of the grid measured 41 mm so as to facilitate section analysis. The ordinates range from Y = 0 to Y = 30. This grid was designed using SketchUp software (Trimble, Sunnyvale, CA, USA) and exported in .3ds format. 

**Figure 2:**
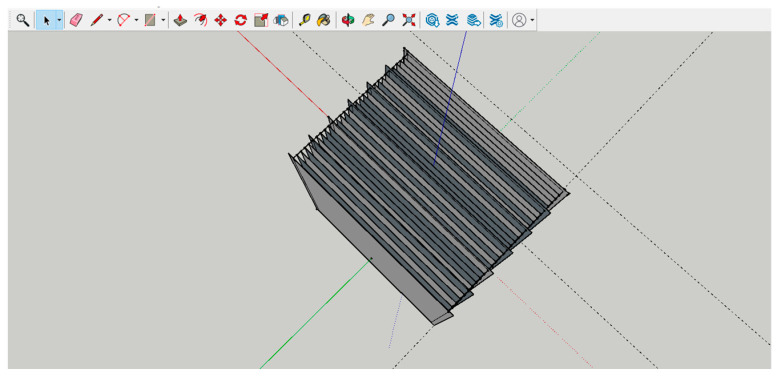
3D Grid.

A single impression at a time was uploaded to Geomagic Control X, then the 3D grid was imported (Figure 2). Using the “Plane” function, 39 orthogonal planes were created on the X-axis and parallel to the Y-axis, 1 mm apart, so that abscissae with coordinates from X = 0 to X = 38 were obtained. The dimensions of the impressions and the number of planes along the X axis, as previously described, were chosen so that the two cusps were crossed in the middle by the section plane.

To identify the abscissae, the 2D comparisons of the report were taken as a reference point, where the 2D1 comparison had a value of X = 0 in correspondence to Plane No. 1.

**Figure 3:**
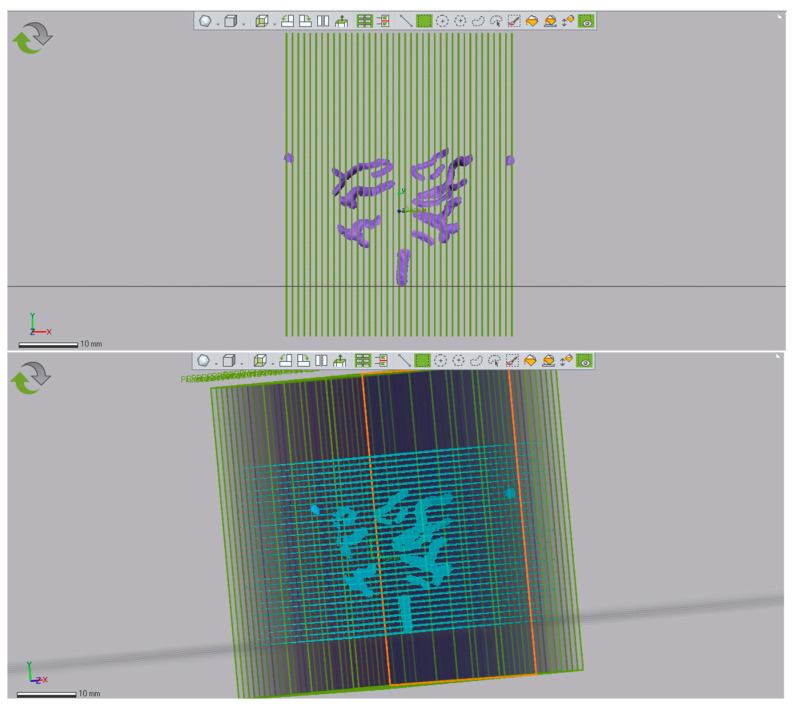
Front and side views on Geomagic Control X.

Using the “multiple 2D compare” tool, 39 sagittal sections were made for each impression (Figure 3). The following commands were selected for all the impressions: Basic Plane (Plane No. 1), No. of Instances 39, Distance 1 mm.

**Figure 4:**
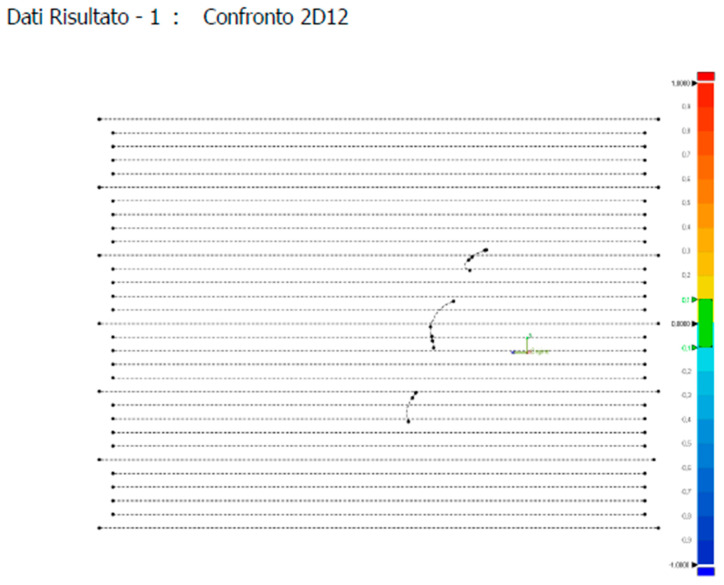
Example of a section.

Finally, through the “Report” tool, all sections were downloaded and then printed (Figure 4).

All coordinates of points that intersected with the grid, or were tangent to it, were identified and uploaded to an Excel spreadsheet, which was different for each sex. All the coordinates that were common among the male subjects and present in more than 50% of the cases were identified, and the same was done with the female subjects. The coordinates present in 50% of the cases in both groups were defined as Common Traits (CT); those present in more than 50% of the cases in only one of the two groups were defined as Discriminant Traits (DT).

The coordinates inherent to the palatal cusps of the second premolars were not considered.

The results obtained were analyzed by *p*-Value using the “Data Analysis, Regression” function on Excel and represented on GeoGebra software (version 6.0.794.0., Markus Hohenwarter-Salzburg, Linz, Austria).

## 3. Results

**Figure 5:**
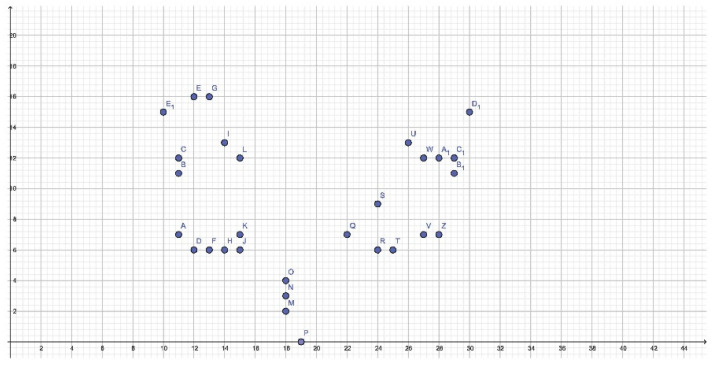
Cartesian plane representation of the DTs in the female group.

2223 impression sections were studied and 145 coordinates that were present with a frequency greater than 50% in one or both groups were obtained: 52 DTs in the male group, 29 DTs in the female group, and 64 CTs. The sum of all coordinates found in the 57 impressions was 9328: 4569 in the male group and 4759 in the female group, the female group seems to have more coordinates than the male group and this is statistically significant (*p* = 0) (Figure 5).

In the female group, the DTs allow us to observe a relatively definite picture of the length of the retroincisal papilla with coordinates slightly placed to the right, with abscissa of X = 18 and apex at X = 19. A possible width of the retroincisal papilla cannot be appreciated as all but one point has an abscissa of X = 18.

The more anterior primary rugae range in the lateral direction along the ordinates from position Y = 6 to Y = 7, while the more posterior ones have less constant coordinates. In the area between the middle third and the posterior third, between the second premolars and the first molars, two DTs can be observed on the right and one on the left where it will probably be possible to find primary or secondary rugae. The DTs in the female group show no statistically significant difference from the same coordinates in men (*p* = 0.832) (Table 1 and Table 2).

**Figure 6:**
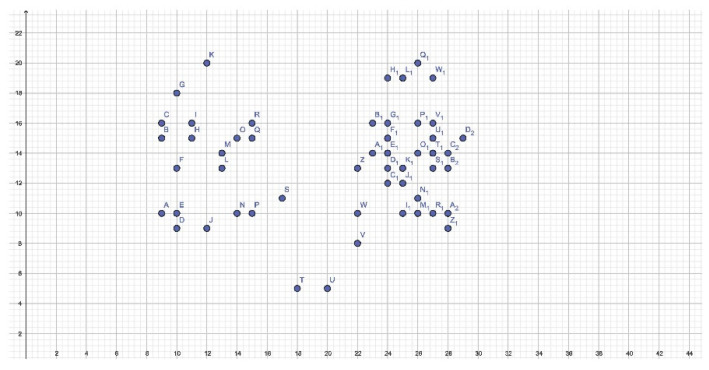
Cartesian plane representation of the DTs in the male group.

In men, the picture is not as unambiguous and defined as in women (Figure 6). In fact, it is shown to be much more diffused. It would seem that the base of the retroincisal papilla is well-defined at the expense of the remaining coordinates of the same, whose frequency is less than 50% and therefore not taken into account.

On the left, an important number of DTs are observed, particularly in the middle third where a pattern comparable to one or more palatal rugae cannot be identified. On the right, on the other hand, immediately posterior to the retroincisal papilla, coordinates with Y = 9 and Y = 10 ordinates, attributable to a rectilinear and/or curved pattern, are observed. In the middle and posterior thirds of the right hemilateral, the highlighted points are arranged so diffusely that there is little definable pattern other than a wavy or curved one (Table 3). This is in line with the results of some studies that affirm that men have more palatal rugae.

**Figure 7:**
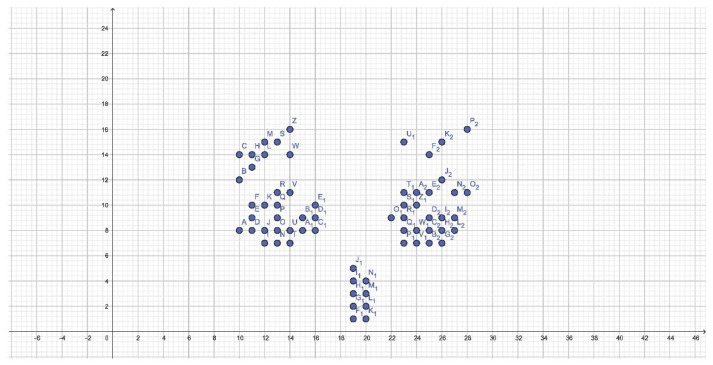
Cartesian plane representation of CTs.

In contrast to the females, males had a higher frequency of palatal rugae in the posterior third, beyond the Y = 16 ordinate. Furthermore, DTs were positioned slightly more to the right than in females. Statistically significant differences were observed here compared to the same coordinates in the female group (*p* = 0.018) (Figure 7).

Among the CTs, the definition of the retroincisal papilla can be noted. As can be expected, its apex is highly variable.

The first and second palatal rugae show a consistent thickness. However, it is not possible to identify whether the pattern is divergent or convergent. In the posterior third of the right hemilateral, it can be seen that the rugae can be curved and directed both anteriorly and posteriorly, while on the left a possible curved rugae with posterior concavity can be identified.

Interestingly, in the posterior third, the points beyond the Y = 16 ordinate occur with a frequency greater than 50% only in males.

Finally, there is a constant distance both on the right and on the left of the median palatine raphe. This is not observed in the graphical representations of DTs, where males have a possible origin of palatal rugae shifted to the left, whereas females do not. No statistically significant differences are observed in the frequency of these coordinates in both sexes (*p* = 0.056).

## 4. Discussion

In the last decade, there has been some interest in palatal rugae as a possible means to identify an individual. To identify an individual accurately, a database would be required, as is the case with fingerprints, and to date, this is not available.

The classic palatal rugoscopy studies the morphology of the palatine rugae and their dimensions (primary or secondary rugae) to establish the identity of the person [3].

Unlike other studies, which mainly evaluated the distance between rugae or their morphology, this study aimed to identify coordinates so as to identify possible DTs between the two sexes by studying their sections, this approach makes it difficult to compare the data obtained with other works. The results obtained, although not all statistically significant, show that women present much more defined coordinates than men and that there may be differences between the two sexes related to the mesial portion of the rugae with respect to the median palatine raphe. It also highlights how, in the posterior third of the palate, men may present palatal rugae more frequently.

The presence of a statistically significant result in the male group suggests that in future, if confirmed by other studies, the characteristics found may be used as a sex discriminator in victim identification. However, the need for a simple and reliable method so as to reduce possible operator-dependent errors should be emphasized.

There exists the possibility of superimposing the impressions onto the DTs obtained from the male group, resizing them as was done in this study. This method, however, would be too specific, with the risk of obtaining a large number of false negatives. Moreover, no statistically valid data was obtained from the female group for the evaluation of any doubtful results.

Ultimately, given the stringent criteria of this study, it is uncertain whether intraindi-vidual variables could vary rugae morphology so much as to make the result incorrect as in extractions, orthodontic treatments, palate expansion [5].

From an anthropological point of view, the focus has been on the identification of ethnicity and sex. Up till now, studies in literature have been based on the number of rugae and their morphology, but not on common punctiform characteristics that may distinguish one sex from another.

We found significant differences in male TDs when comparing the same coordinates in the female group but we but we have not been able to find a variable that allows us to discriminate between sexes. In according with our study statistically significant differences in palatal rugae patterns have been found between two ethnic groups in Nigeria, but the possibility of being able to distinguish between sexes has not been demonstrated [6]. The same was confirmed by a study on the Iranian population [7]. Other studies carried out on the Sudanese and Portuguese population state that a certain morphology of palatal rugae can be used to identify a certain geographical area, and that the dysmorphism between the male and female sex is slight and can hardly be applicable as a marker in forensic odontology in the Sudanese population, in which there appears to be little dysmorphism between the two sexes [8,9].

In a study conducted in India in 2019, no differences were observed in three different southern populations. However, it was noted that the curved pattern was more common in women than in men, where there was a higher incidence of straight rugae. The wavy pattern was equally distributed among the two sexes [10]. Out of 100 Gujarati subjects, no difference was found in the two sexes in either metric or morphological parameters [11]. The classification of Thomas et al., however, was not used.

In a total of 50 Tibetan and 50 Indian subjects, it was observed that the Tibetan ethnic group had significantly fewer palatal rugae, with a predominance of the curved pattern, while the Indians showed a predominantly wavy pattern [12]. Also in India, among 500 subjects from five different populations, the predominant curved pattern was identified [13].

In a further study on the Iranian population to determine sex, a statistically significant predominance in the number of curved rugae was described in women, particularly on the right side, with a consistent number of primary rugae in both sexes [14].

In Kerala (South India), out of a total of 100 individuals, a statistically significant greater number of rugae was noted in women, while in men the circular pattern was prevalent [15]. Similar to the previous study, also on the Indian population, a greater number of palatal rugae was observed in women than in men. However, males showed a greater number of wavy rugae, while in women the straight pattern was prevalent [16]. Contrary to what has been stated so far, Shreesh Mhatre V. et al. noted that the total number of rugae was greater in men than in women, confirming that the wavy pattern was more prevalent in men rather than in women [17]. These last two studies, looking at our graphical representations of the coordinates, would seem to agree with our results. A prevalence of divergent rugae in women and convergent rugae in men is suggested by one study [18], which is in contrast with an analysis performed on the Meerut population, in which contrasting data are observed, along with a circular pattern more present in men than in women [19]. The same result was observed on a sample of 100 Indian children. A higher frequency of convergent and divergent rugae was observed in males and females respectively, further confirming that men have a statistically significant prevalence of circular rugae [20]. However, in a further study of 130 Iranian subjects, the divergent pattern is statistically greater in men than in women [21].

The use of digital technology proved to be of greater assistance than analogic means in the processing of the impressions, although several hurdles must be still be overcome. We do not know whether the simultaneous use of multiple software affected the accuracy of the study, while the analysis of the sections is operator dependent.

It should also be considered that there is no firm data on the reliability of scanners in forensic odontology, so caution should be taken when using this equipment [22,23,24]. An important point to make regards the manipulation of data, which could be performed with intentions that are anything but ethical.

An additional strength of digital technology is the ability to optimize time in data analysis.

It is certain that the assistance of digital technology in forensic odontology can be relevant, but equally relevant is the transfer of data from a physical structure to a computerized one, as in the case of digital scanners, where the data detected is not necessarily identical to the real data [25,26]. The use of increasingly sensitive instruments, at the expense of specificity, can certainly lead to false positives, as does the use of the .stl format, given that it lacks colour, reducing the operator’s ability to be able to accurately identify palatal rugae.

To date, there is no software dedicated to forensic odontology. In the various studies observed in literature, software dedicated to engineering and therefore useful in laboratory conditions or applications dedicated to photography are often used. It follows that no software has all the features suitable for carrying out studies such as this one. Resorting to expedients runs the risk of creating errors in the transition from one program to another, due to the use of different programming languages [27] or implementations. On the other hand, if a software makes an error, it is systematically repeated and becomes more predictable.

To date, these issues, along with the small size of the sample group taken as the object of the study, do not allow these results to be widely applied with reliability.

## 5. Conclusions

This study highlighted how there may be topographical differences in the area of palatal rugae from an anthropological point of view in the two sexes [28]. It is observed that in men, palatal rugae extend more frequently in the posterior third than in women and that they exhibit more possible discriminatory features than women. Women manifest more uniformly defined discriminatory coordinates than men but, conversely, these are significantly fewer in number than in the other sex.

It is observed that the statistical result in the male group is significant compared with DTs in the female group and CTs.

The limited number of samples, the frequency of the coordinates considered (>50%) and the lack of an otherwise reliable statistical result are the main limitations of the study. Although statistically significant differences (*p* = 0.018) were found in the male TDs compared to the same female coordinates, it was still not possible to identify a discriminant between the two sexes.

It is certain that digital technology may one day be a valuable support for the forensic odontologist but to date, the lack of dedicated and certified programs limits its reliability.

## Figures and Tables

**Table 1:** Female DTs and frequency (%F).

X;Y	% F	X;Y	% F	X;Y	% F	X;Y	% F
10;15	53.33%	14;13	56.67%	18;4	63.33%	27;12	56.67%
11;11	53.33%	14;6	56.67%	19;0	63.33%	27;7	56.67%
11;12	53.33%	15;12	60.00%	22;7	53.33%	28;12	56.67%
11;7	56.67%	15;6	53.33%	24;6	56.67%	28;7	53.33%
12;16	56.67%	15;7	63.33%	24;9	56.67%	29;11	53.33%
12;6	56.67%	18;2	63.33%	25;6	56.67%	29;12	60.00%
13;16	53.33%	18;3	63.33%	26;13	53.33%	30;15	56.67%
13;6	56.67%						

**Table 2:** Male DTs and frequency (%M).

X;Y	%M	X;Y	%M	X;Y	%M	X;Y	%M
10;10	66.67%	15;15	51.85%	24;15	51.85%	27;13	59.26%
10;13	55.56%	15;16	51.85%	24;16	51.85%	27;14	70.37%
10;18	51.85%	17;11	51.85%	24;19	55.56%	27;15	70.37%
10;9	51.85%	18;5	51.85%	25;10	70.37%	27;16	62.96%
11;15	62.96%	20;5	66.67%	25;12	55.56%	27;19	55.56%
11;16	55.56%	22;10	55.56%	25;13	59.26%	28;10	59.26%
12;20	51.85%	22;13	51.85%	25;19	55.56%	28;13	59.26%
12;9	74.07%	22;8	55.56%	26;10	59.26%	28;14	62.96%
13;13	55.56%	23;14	59.26%	26;11	70.37%	28;9	62.96%
13;14	74.07%	23;16	55.56%	26;14	55.56%	29;15	55.56%
14;10	62.96%	24;12	55.56%	26;16	55.56%	9;10	51.85%
14;15	51.85%	24;13	51.85%	26;20	51.85%	9;15	51.85%
15;10	51.85%	24;14	51.85%	27;10	66.67%	9;16	55.56%

**Table 3:** Common coordinates in both sexes.

X;Y	X;Y	X;Y	X;Y	X;Y	X;Y	X;Y	X;Y
10;12	12;10	13;7	15;8	19;4	23;11	24;8	26;7
10;14	12;14	13;8	15;9	19;5	23;15	25;11	26;8
10;8	12;15	13;9	16;10	20;1	23;7	25;14	26;9
11;10	12;7	14;11	16;8	20;2	23;8	25;7	27;11
11;13	12;8	14;14	16;9	20;3	23;9	25;8	27;8
11;14	13;10	14;16	19;1	20;4	24;10	25;9	27;9
11;8	13;11	14;7	19;2	22;9	24;11	26;12	28;11
11;9	13;15	14;8	19;3	23;10	24;7	26;15	28;16

## Data Availability

Data can be requested to alessandro.scardina@unipa.it.
